# Differential Item Functioning Related to Age in the Reading Subtest of the Test of Functional Health Literacy in Adults

**DOI:** 10.1155/2013/654589

**Published:** 2013-09-08

**Authors:** Raymond L. Ownby, Drenna Waldrop-Valverde

**Affiliations:** ^1^Department of Psychiatry and Behavioral Medicine, Nova Southeastern University, Fort Lauderdale, FL 33318, USA; ^2^Emory University, Atlanta, GA 30322, USA

## Abstract

Differential item functioning (DIF) occurs when items in a measure perform in ways that are different for members of a target group when the different performance is not related to the individual's overall ability to be assessed. DIF may arise for a number of reasons but is often evaluated in order to ensure that tests and measures are fair evaluations of a group's abilities. Based on observations when administering the test, we developed the hypothesis that some items on the reading comprehension subtest of the Test of Functional Health Literacy (TOFHLA) might be differentially more difficult for older adults and the elderly due to its use of the cloze response format, in which the participant is required to determine what word, when placed in a blank space in a sentence, will ensure that the sentence is intelligible. Others have suggested that the cloze response format may make demands on verbal fluency, an ability that is reduced with the increasing age. Our analyses show that age-related DIF may present in a nearly one-half of reading comprehension items of the TOFHLA. Results of this measure in older persons should be interpreted cautiously.

## 1. Introduction

Health literacy has assumed increasing importance over the past decade as research has continued to accumulate showing that patients' levels of it have important relations to their health, use of health services, and health outcomes [1, 2]. Health literacy is defined as “*… the degree to which individuals have the capacity to obtain, process, and understand basic health information and services needed to make appropriate health decisions* [3].” It has been related to a number of variables reflecting patients' ability to obtain and use information to reach their desired state of health, including use of preventive health services, indices of disease control such as glycosylated hemoglobin in diabetes, risk for hospitalization, and even increased likelihood for death [1, 4].

 One especially important finding in health literacy research has been the fact that racial and ethnic minorities and the elderly perform at lower levels on several measures of health literacy compared to the general population [5, 6]. One widely cited study, for example, was the National Assessment of Adult Literacy (NAAL) which included a health literacy scale [5]. The study, based on a nationally representative sample, showed that blacks, Hispanics, and the elderly had lower levels of health literacy on the NAAL health literacy scale. Studies with other measures, including the widely used Test of Functional Health Literacy in Adults (TOFHLA) [7], have also found similar differences. Given the link between health literacy and health status and the common finding of disparities in health among racial and ethnic minorities, several authors have suggested that differences in health literacy may be a factor in health disparities [6].

 Although studies have often treated health literacy as a unitary characteristic of the persons assessed, studies have used a number of different measures to assess it [4]. It is not clear, however, whether various measures of health literacy assess the same abilities and skills. The TOFHLA, for example, includes two subtests that assess reading comprehension and numeracy skills. An issue that may limit the usefulness of the TOFHLA is the response format in the reading comprehension subtest. The TOFHLA uses the cloze procedure [8] to assess reading comprehension. In this approach, comprehension is tested by asking the person evaluated to demonstrate their understanding by supplying a word missing in a sentence (e.g., “The sky is —”). This strategy may create items that are differentially more difficult for older persons as it taps abilities known to decline with increasing age [8, 9].

Another widely used measure, the Rapid Estimate of Adult Literacy in Medicine, or REALM [10], assesses health literacy only regarding the person's ability to read a list of health-related words aloud. Other measures of health literacy may evaluate still other abilities using other response formats. The Rapid Estimate of Adult Literacy in Medicine, or REALM [10], only assesses health literacy regarding patients' ability to correctly pronounce a series of health-related words (e.g., anatomical terms and the names of diseases and condition) and thus does not directly assess their ability to understand what they read. The REALM does not assess numeracy skills, consistently shown to be an important aspect of health literacy [11]. The Newest Vital Sign [12] only assesses patients' comprehension of a single food label, and thus it only taps a very narrow range of skills. Further, the psychometric characteristics of most measures are not well known, as noted by Jordan et al. [13]. One important task for health literacy researchers is to better understand the currently available measures of health literacy and to address concerns about scale characteristics in developing new measures [14].

 In a previous study, we used the TOFHLA with elderly patients who were being treated with medications for memory problems [15, 16]. In pilot testing of the study assessment battery, it became apparent that many elderly patients had difficulty with the cloze format of the TOFHLA reading comprehension, appearing to not understand the task even after multiple explanations and finding it difficult to produce responses even when able to choose from multiple available choices. By contrast, younger persons commonly have little or no difficulty with the response format. These observations led us to evaluate the possibility that the TOFHLA response format might be differentially more difficult for older compared to younger individuals.

 Other authors have suggested that the cloze format may be difficult for older adults due to its demands on cognitive abilities known to decline with increasing age, including verbal fluency working memory and psychomotor speed [17]. Further, Ackerman et al. showed that cloze performance modified the relation between age and general cognitive ability [8]. If cloze items are in fact differentially more difficult for older adults due to changes in their basic cognitive abilities, then a health literacy measure that uses this response format might produce results suggesting that elders' health literacy skills are lower than they actually are. One strategy to evaluate this possibility is to assess whether the items are associated with *differential item functioning* (DIF) [18]. DIF is said to exist for a particular item in a measure when its difficulty is not the same for individuals of equal ability. In the case of health literacy measures like the TOFHLA that use the cloze procedure [8], the result would be that some items would be more difficult for older individuals than for younger individuals with same overall health literacy ability, not because of actual differences in health literacy but because the item requires a cognitive ability (e.g., verbal fluency) that is lower in the older individuals. The item would thus tap two abilities (health literacy and verbal fluency) while ostensibly assessing only one (health literacy). Since the second ability differs between the two groups, the item will appear to be more difficult for older individuals, but not because they actually have lower health literacy. The purpose of this paper was thus to evaluate whether the cloze items on the TOFHLA presented evidence of age-related DIF. We hypothesized that the response format of the measure would result in evidence of age-related DIF.

## 2. Method

### 2.1. Participants

 Data for this study were drawn from a study of cognition and medication adherence in persons treated for HIV [19]. Participants were recruited from several local clinics in South Florida, USA, and were referred by healthcare providers or as a result of their having seen flyers that publicized the study. All were 18 years of age or older and were judged as requiring treatment for HIV infection. Participants were screened for serious neurological or psychiatric impairment and indicated that they had not used illicit drugs during the past 12 months. The full testing procedure required no more than 2 hours for completion, and subjects were paid $50 for their participation.

### 2.2. Measures

As part of a battery of measures, the reading comprehension portion of the TOFHLA was administered. This measure comprises three health-related paragraphs of increasing reading difficulty, beginning with instructions on how to prepare for a radiographic study and concluding with an informed consent for a surgical procedure. Words are removed from sentences with a blank substituted, and possible correct options are listed below each blank. The total number of responses for the all paragraphs is 50. Participants were tested according to the standard directions for the measure [7] and were given 20 minutes to complete the questions. Their responses were categorized as right or wrong according to the test's administration instructions [7].

### 2.3. Procedures

 Sample sizes required for stable estimates via parametric item response theory (IRT) are large. Most experts suggest that sample size should be in the range of 1,000 [18]. Because of our small sample size, data analyses were completed using a nonparametric item response theory (IRT) strategy using the TestGraf software (http://www.psych.mcgill.ca/misc/fda/downloads/testgraf/), a package that is freely available for download [20]. In addition to providing nonparametric IRT plots of the relation of participants' overall ability to their probability of obtaining a correct answer, this software package calculates a measure of overall DIF, *beta*, for each item. Based on extensive simulation modeling, Zumbo and Witarsa [21] have provided critical values for the beta statistic in relation to various sample sizes. These authors also show that the use of these critical values has considerably better power for detecting the presence of known DIF than the better-known strategy of calculating Mantel-Haenszel chi-square values. We also used jMetrik, a freely available software package for item analysis (http://www.itemanalysis.com/), to estimate item difficulties, standard deviations, and discriminations (defined as the correlations of each item with the total scale score). 

We divided our sample into two groups, those with ages less than and those with ages equal to or greater than 45 years. This cut point was chosen as it provided reasonably similar sample sizes for each group and lies in the age range related to both evidence of cognitive aging [22] and lower levels of health literacy [5]. Items that exceeded the critical value of beta for our sample size as reported by Zumbo and Witarsa [21] for a probability of less than 0.01 were flagged for examination of item plots and are marked in our results below.

## 3. Results

 Statistics providing a characterization of the sample are presented in Table 1. The majority of participants were men and black, and there was a wide range of age and education in the sample.

**Figure 1:**
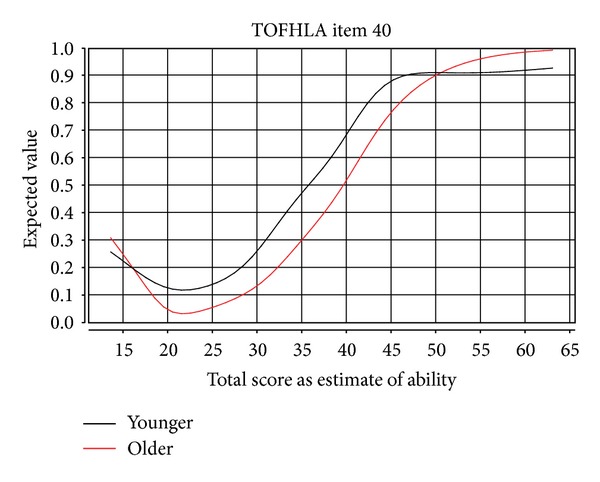
Item curve illustrating DIF between younger and older persons.

 Results of item analyses are presented in Table 2. Items with beta values greater than the *P* < 0.01 cut point provided by Zumbo and Witarsa are italicized [21]. It can be seen that 24 out of the 50 items show significant age-related DIF. The impact of age-related DIF on test performance is illustrated in Figure 1, based on analyses for item 40 in paragraph C of the TOFHLA reading comprehension test. It shows item curves for younger and older individuals; each plots the probability of someone obtaining a correct answer on question 40 (left axis, ranging from 0 to 1) and the participant's underlying general health literacy ability estimated as their total score on the measure. The plot includes lines for younger participants (marked with a 1) and older participants (marked with a 2). If an item does not present DIF, the lines should approximately coincide, and the beta value should be near 0. A consistent distance between the lines suggests that the item is more or less difficult for members of one group or another. As illustrated in Figure 1, older individuals must have a higher level of ability to obtain a correct answer than younger persons do. The impact of age-related DIF would thus cause older individuals to have lower overall scores because of the relatively greater difficulty of these items.

## 4. Discussion

 Results of these analyses suggest the existence of substantial age-related DIF in the reading comprehension subtest of the TOFHLA. To the best of our knowledge, this is the first study to evaluate at an item level the influence of age on TOFHLA scores. Based on our observations of participants in an earlier study, we investigated the possibility that the response format of the TOHFLA might have an influence on older adults' performance independent of their actual levels of health literacy. Our results suggest that this may be the case. The implication of this finding is that at least a portion of the difference in health literacy associated with age on the TOFHLA may be the result of DIF rather than actual differences in health literacy.

 It should be noted that some studies have not found age-related differences in health literacy when using a measure that does not use the cloze response format (Rapid Estimate of Health Literacy in Medicine or REALM [10]). One study, for example, administered both the S-TOFHLA and the short form of the REALM in adults with diabetes [23]. While the expected age-related differences emerged for the reading section of the S-TOFHLA, none were found for the REALM. Shigaki et al. [24] compared the REALM and another measure, the Newest Vital Sign (NVS) [12]. In this study, age differences emerged for the NVS (which requires that patients generate answers) but not for the REALM. In a large sample of persons with a wide range of educational and health backgrounds, Sudore et al. [25] also failed to find age-related differences in health literacy as assessed by the REALM. 

 Limitations of this study should be acknowledged. Our sample included only persons treated for HIV infection, potentially limiting the extent to which these findings can be generalized to other populations of older adults. Although our participants may have had HIV-related cognitive deficits that could have affected their performance on the S-TOFHLA, it is also likely that they would have had age-related changes in cognitive function. The dual effects of HIV infection and aging on cognitive function (presumably the basis for finding DIF on the S-TOFHLA) are difficult to distinguish; studies of the issue have suggested that both aging and HIV have an impact on cognition [26] while at least one study did not find a relation [27]. Older participants might be differentially more susceptible to fatigue during testing procedures. Since the TOFHLA questions were embedded in a larger battery of cognitive measures, this might have affected older persons' responses. We note that the entire battery is only required for at most 2 hours and that our participants were all community-dwelling and ambulatory, reducing the likelihood of serious fatigue affecting their responses. This possibility, however, cannot be ruled out.

While it thus might appear that age-related deficits in health literacy may be related to the response format of the measure used to assess it, it must be noted that other measures have found age-related differences in health literacy. Although (due to concerns for test security that prohibit revealing actual items) it is difficult to know the precise format of responses on the measure, the National Assessment of Adult Literacy found significant deficits in health literacy among older adults. Haun et al. found age-related differences in performance on the S-TOFHLA and a self-report measure of health literacy, the BRIEF [28], but not on the REALM. It may be reasonable to conclude, as have others, that it may be important to consider task demands and purpose when selecting a health literacy measure for a particular purpose [29]. These results thus further support others' observations of the variable relations of common measures of health literacy with age. Given the evidence of age-related DIF on a substantial number of items in the reading comprehension subtest of the TOFHLA, it would appear prudent to be cautious in interpreting the significance of age-related deficits in health literacy when it is assessed using the TOHFLA or S-TOFHLA.

## Figures and Tables

**Table:** (a)

Gender	119 men, 88 women
Hispanic	22

Race	

Non-Hispanic White	9 (4.3%)
Hispanic	21 (10.1%)
Black	174 (83.7)
Native American	1 (0.5%)

**Table:** (b)

Continuous variables			
Variable	Range	Mean	Standard deviation
Age	23–67	45.0	8.45
Education (years)	5–20	11.61	2.18
TOFHLA reading comprehension score	2–50	38.51	7.77

**Table 2:** Item difficulties, discriminations, and betas.

Item	Difficulty	SD	Discrimination	Beta
A1^a^	0.880	0.326	0.398	***0.087***
A2	0.918	0.275	0.520	0.047
A3	0.809	0.394	0.504	***0.116***
A4	0.924	0.267	0.510	0.049
A5	0.940	0.238	0.499	0.032
A6	0.967	0.179	0.163	0.012
A7	0.874	0.332	0.549	0.025
A8	0.880	0.326	0.444	0.058
A9	0.858	0.350	0.386	***0.085***
A10	0.880	0.326	0.562	0.026
A11	0.902	0.299	0.472	0.063
A12	0.951	0.217	0.444	0.060
A13	0.902	0.299	0.417	0.052
A14	0.863	0.344	0.395	0.062
A15	0.842	0.366	0.391	***0.073***
B16	0.934	0.248	0.396	0.054
B17	0.924	0.267	0.473	0.044
B18	0.951	0.217	0.431	0.058
B19	0.645	0.480	0.393	***0.094***
B20	0.902	0.299	0.390	0.057
B21	0.721	0.450	0.504	***0.115***
B22	0.836	0.371	0.427	0.044
B23	0.934	0.248	0.369	0.036
B24	0.634	0.483	0.361	***0.170***
B25	0.809	0.394	0.464	***0.071***
B26	0.809	0.394	0.451	***0.099***
B27	0.863	0.344	0.601	***0.082***
B28	0.913	0.283	0.470	0.051
B29	0.820	0.386	0.543	0.034
B30	0.754	0.432	0.660	***0.109***
B31	0.798	0.403	0.520	***0.072***
B32	0.814	0.390	0.520	***0.118***
B33	0.869	0.339	0.526	0.054
B34	0.426	0.496	0.394	***0.151***
B35	0.874	0.332	0.666	0.062
B36	0.863	0.344	0.641	0.052
C37	0.863	0.344	0.570	***0.079***
C38	0.743	0.438	0.707	0.031
C39	0.579	0.495	0.512	***0.091***
C40	0.579	0.495	0.522	***0.146***
C41	0.448	0.499	0.529	0.050
C42	0.710	0.455	0.627	0.051
C43	0.628	0.485	0.633	***0.100***
C44	0.678	0.469	0.630	***0.074***
C45	0.251	0.435	0.346	***0.183***
C46	0.481	0.501	0.621	***0.110***
C47	0.197	0.399	0.244	***0.074***
C48	0.519	0.501	0.535	0.048
C49	0.568	0.497	0.564	***0.090***
C50	0.628	0.485	0.694	***0.070***

^a^Letter prefixes before items numbers denote from which of the three test paragraphs the item is drawn.
